# Anti-Oxidative Activity of An Aqueous Suspension of Commercial Preparation of The Mushroom *Coprinus comatus*

**DOI:** 10.3390/molecules15074564

**Published:** 2010-06-24

**Authors:** Mira Popović, Saša Vukmirović, Nebojša Stilinović, Ivan Čapo, Vida Jakovljević

**Affiliations:** 1 Department of Chemistry, Faculty of Sciences, Trg Dositeja Obradovica 3, 21000 Novi Sad, Serbia; 2 Department of Pharmacology, Toxicology & Clinical Pharmacology, School of Medicine, Hajduk Veljkova 3, 21000 Novi Sad, Serbia; E-Mail: sasavukmirovic@neobee.net (S.V.); 3 Department of Histology, School of Medicine, Hajduk Veljkova 3, 21000 Novi Sad, Serbia; E-Mail: capo.ivan@gmail.com (I.C.)

**Keywords:** *Coprinus comatus*, antioxidant, hepatoprotective

## Abstract

In this study the effects of an aqueous suspension of a commercial preparation of the mushroom *Coprinus comatus* on oxidative stress induced in rats by alloxane and carbon tetrachloride was examined. The effects were estimated from changes in the biochemical parameters (xanthine oxidase, glutathione peroxidase and catalase activity, reduced glutathione content, and extent of lipid peroxidation) of liver homogenate as well as histological changes in the liver of the rats treated with alloxane and carbon tetrachloride. Two screening doses of alloxane sufficient to induce diabetes in rats did not have any significant effect on the examined biochemical parameters of liver homogenate or on the cytoarchitectonics of liver cross-sections. Treatment with carbon tetrachloride resulted in a significant increase in the intensity of lipid peroxidation and peroxydasis activity, as well as with decrease in catalase activity. Certain changes in liver cross sections were detected, such is lymphocyte infiltration of dilated sinusoid capillaries. Administration of *Coprinus comatus* suspension thus showed antioxidative potential, evidenced by an increase of antioxidative status of liver homogenate and prevention of histological changes in liver cross sections.

## 1. Introduction

Mushrooms (Fungi) are one of the kingdoms in the Eukaryota domain. Since they do not have chloroplasts, mushrooms are classified as heterotrophic organisms. They can also be saprophytic, which determines them as key factors in the last phases of degradation of live and dead organic matter. Various enzymatic reactions occur in mushrooms what gives them significant metabolic potential. Mushrooms adapt with ease to habitat changes, and they synthesize different secondary bio-molecules, some of which are toxins, and others which are therapeutic. It was found that mushrooms, as well as other aerobic organisms, have developed anti-oxidative activity, which improves their survival potential. 

Because of the growing exposure of humans to various environmental pollutants, there has been increased interest in mushrooms as potential sources of antioxidants in human diet [1,2,3,4,5,6]. This benefit of mushrooms has been exploited in the Far East for thousands of years, while in Western cultures people became aware of this fact in last two decades or so, when research in edible and therapeutic mushrooms intensified [7,8]. The chemical composition of mushroom supplements is of special interest, since the chemical profile sometimes differs within the same mushroom species. Among other parameters, it is the habitat which influences chemical profile, and makes the mushroom suitable or not for the diet or pharmaceutical industry [1,9,10]. 

In this research a commercial preparation of the mushroom *Coprinus comatus*, which is assumed to have nutritive as well as curative potential, was used [11]. *Coprinus comatus* is an edible mushroom species which produces an inky liquid full of spores, and it is widespread in the Northern Hemisphere, where it usually grows in meadows or along dirt roads [12]. 

The aim of this study was to determine the anti-oxidative activity of a preparation of the mushroom *Coprinus comatus* by itself and in combination with prooxidants (alloxan and carbon tetrachloride) on the following liver biochemical parameters: xanthine oxidase, glutathione peroxidase and catalase activity, reduced glutathione content, and extent of lipid peroxidation. The influence of this preparation on liver cytoarchitectonics, with and without prooxidants, was also examined. Sabo *et al.* [13] had previously described certain pharmacodynamic activity of this commercial preparation in the same experimental model. 

## 2. Results and Discussion

### 2.1. Biochemical parameters of rat liver homogenate

Table 1 shows the measured biochemical parameters of the rat liver homogenate. As shown in this Table, compared to the control group liver enzyme levels did not change in rats treated with *Coprinus comatus *aqueous suspension, except for GSH content, which was significantly increased. This increase can be attributed to the treatment with *C. comatus* aqueous suspension, which leads to the conclusion that *C. comatus* possesses protective effects. 

**Table 1:** Effect of *Coprinus comatus* aqueous suspension on liver biochemical parameters

	LPx	XOD	CAT	Px	GSHPx	GSH
**Control**	0.580 ± 0.093	1.958 ± 0.181	0.285 ± 0.053	1.876 ± 0.159	10.469 ± 1.627	0.483 ± 0.087
***C. comatus***	0.567 ± 0.053	2.097 ± 0.294	0.216 ± 0.019	1.80 ± 0.160	12.937 ± 1.285	0.711 ± 0.108^*^
**Alloxan**	0.741 ± 0.106	2.337 ± 0.31	0.185 ± 0.034^*^	2.772 ± 0.180^*^	14.814 ± 1.822^*^	0.414 ± 0.065
**Alloxan +*C. comatus***	0.530 ± 0.108	2.441 ± 0.229	0.254 ± 0.029	2.381 ± 0.172^*,a^	11.925 ± 1.856	0.399 ± 0.076
**CCl_4_**	1.053 ± 0.058^*^	2.74 ± 0.219^*^	0.172 ± 0.011^*^	3.72 ± 0.176^*^	12.478 ± 1.716	0.387 ± 0.072
***C. comatus* + CCl_4_**	0.727 ± 0.04^*,a^	2.58 ± 0.180^*,a^	0.294 ± 0.051	2.90 ± 0.194^*,a^	11.512 ± 0.879	0.765 ± 0.081^*^

^*^p < 0.05 compared to Control, ^a ^p < 0.05 compared to *C. comatus*, t-test, n = 6; x±SD. Content of GSH is expressed in nmol GSH/mg of protein. Activities of XOD, GSH-Px, GSHR, Px, CAT are expressed in nmol/mg of protein min^-1^. Content of LPx is expressed in nmol malondialdehyde/mg of protein.

Two doses of alloxan, which induced diabetes and destroyed pancreatic beta cells, as reported by Sabo *et al.* [13], increased substantially the activity of Px and GSHPx, and at the same time decreased CAT activity, also in a statistically significant manner. Dominant activity of peroxidases could be the reason for the decreased CAT activity. Treatment with C. comatus aqueous suspension followed by two doses of alloxan resulted in a statistically significant increase in Px activity, although the increase was smaller than in the group treated with only two doses of alloxan. Other parameters were not significantly changed. Alloxan and its metabolites act as cytotoxins due to superoxide anion radical, which is generated in the reduction cycle. Superoxide anion radicals undergo dismutation to hydrogen peroxide, which is the source of the highly reactive hydroxyl radicals formed in the Fenton reaction. The reactive oxygen products increase cytosolic calcium levels and destroy pancreatic beta cells [14]. 

Treatment with one dose of carbon tetrachloride resulted in a statistically significant increase in lipid peroxidation (LPx) and peroxidase activity Px and XOD, as well as in a significant decrease in catalase activity (CAT). Other parameters were not significantly changed compared to control group (Table 1). The administered dose of carbon tetrachloride is used as a prooxidant since it is a liposoluble molecule, easily absorbed regardless of the route of administration. It is absorbed in high percentage in adipose tissue, bone marrow, and especially in liver, where it is metabolized by Cyt P450-2E1 [11,15]. 

Seven day treatment with C. comatus aqueous suspension, followed by one dose of carbon tetrachloride, changed the activity of peroxidase, XOD and lipid peroxidation intensity in a statistically significant manner. Changes were smaller when compared to changes detected in the group treated only with carbon tetrachloride, where measured values were more similar to control values. With these facts in mind we can conclude that C. comatus has protective effects against carbon tetrachloride toxicity, especially because the content of GSH in group C. comatus + CCl4 is almost equal to the content of GSH in group of animals treated only with C. comatus. 

Similar results effects were published by Lee [16], who examined the effects of *Fomes fomentarius* supplementation in streptozotocin-induced diabetic rats. In that study *Fomes fomentarius* extract lowered the serum glucose level, increased glutathione peroxidase activity and significantly lowered superoxide dismutase and catalase activities. 

### 2.2. Liver cross sections

**Figure 1:**
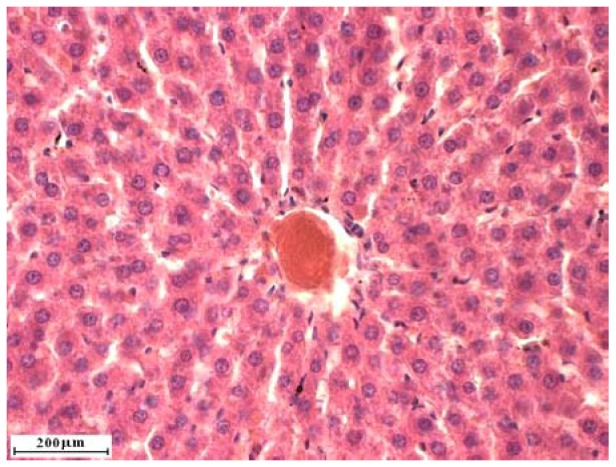
Liver cross section of rats treated with two doses of alloxan.

**Figure 2:**
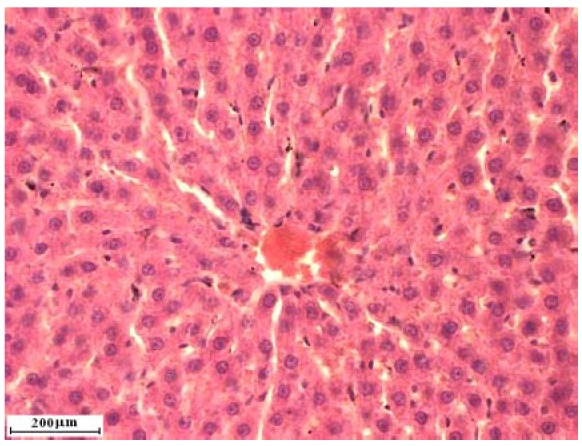
Liver cross section of rats treated with *Coprinus comatus *aqueous suspension.

**Figure 3:**
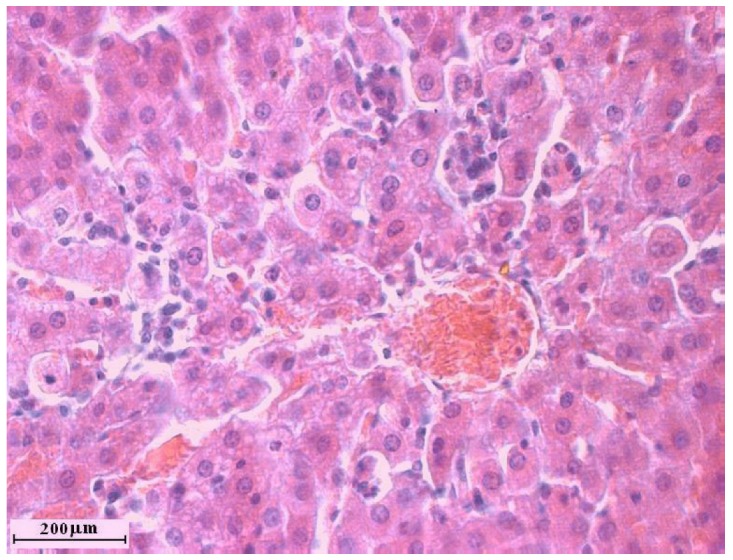
Liver cross section of rats treated with one dose of carbon tetrachloride.

**Figure 4:**
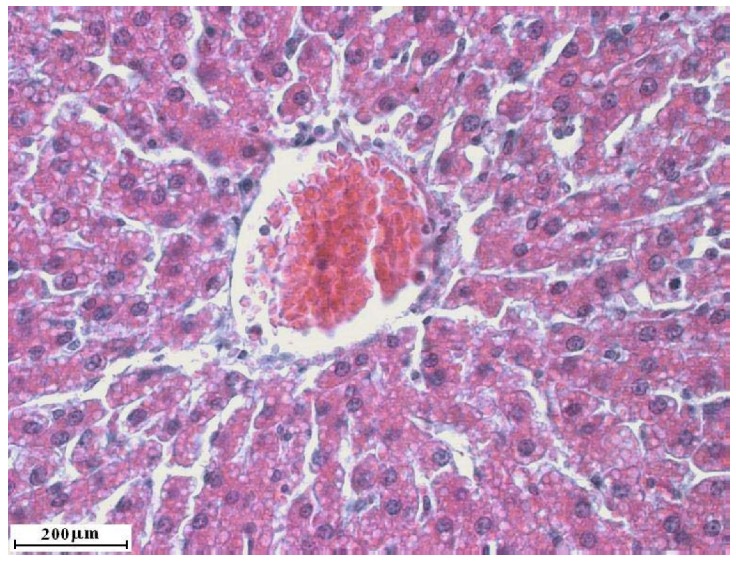
Liver cross section of rats treated with *Coprinus comatus* aqueous suspension for 7 days before one dose of carbon tetrachloride.

Liver cross sections are shown on Figure 1, Figure 2, Figure 3, Figure 4. Analysis of liver cross sections in group of animals treated with two doses of alloxan did not show any changes in liver lobulus cytoarchitectonics. Patohistological changes were not detected in liver tissue (Figure 1 and Figure 2), even though the applied doses are sufficient for diabetes induction. In a study previously published by Kume *et al.* [17] streptozocin, also used for diabetes induction in laboratory animals, caused liver tissue damage. This result suggests that it is better to use alloxan in diabetes induction. 

Treatment with one dose of carbon tetrachloride did not destroy liver lobulus cytoarchitectonics although clear signs of acute hepatitis were detected, such as significant lymphocyte infiltration of the dilated sinusoidal capillaries with a small degree of hepatocyte vacuolar degeneration. Combined treatment with C. comatus followed by one dose of carbon tetrachloride resulted in similar changes, but without lymphocyte infiltration. Such results suggest that seven day treatment with C. comatus suspension before carbon tetrachloride administration resulted in certain protective effects.

## 3. Experimental

### 3.1. Animals

Experiments were carried out on white Whistar laboratory rats of both sexes, with an average body weight of 250-300 grams and ages up to three months. Animal care and all experimental procedures were conducted in accordance with the *Guide for the Care and Use of Laboratory Animals*, edited by Commission of Life Sciences, National Research Council, USA. Rats were bred in the vivarium at the Department of Pharmacology, Toxicology and Clinical Pharmacology, Medical Faculty, University of Novi Sad, Serbia. Animals were kept in standard plexiglass cages at a constant 21 ± 1 ºC room temperature and 55% ± 1.5% humidity with standard circadian rhythm (day/night). They were fed with standard laboratory rat feed, produced by the Veterinary Institute in Zemun (Serbia). Rats were randomly divided in control and test groups, each group consisting of six animals. 

### 3.2. Experimental procedures

Aqueous suspension of commercial *Coprinus comatus* powder (100%, product for human use) imported by Rick’s Technology d.o.o. Novi Sad Serbia, www.lekovito.com) was administered to animals in the test groups for seven days, 1.67 g/kg each day, while physiologic solution was given to animals in the control group. Aqueous suspension to test groups, and physiologic solution to the control group were administered each day between 10 and 11 am. On the seventh day, two hours before the beginning of the experiment the last dose was administered orally. Oxidative stress was induced with prooxidant substances, carbon tetrachloride and alloxan in the recommended doses [18]. 

Alloxan (alloxan monohydrate, Sigma Chemicals Co, St Louis, MO, USA) was used for diabetes induction in the laboratory animals. Two doses of alloxan [each dose 100 mg/kg intraperitoneal (i.p.)] were administered, the first dose two hours after the last dose of *Coprinus comatus* suspension, and the second dose 24 hours after the first dose of alloxan. Hyperglycemia was detected 48 hours after the second dose of alloxan, and animals were sacrificed in that period. Carbon tetrachloride (Sigma Chemicals Co, St Louis, MO, USA) mixed with olive oil 1:1 (v/v) was administered to rats (2 mL/kg i.p.) two hours after the last dose of *Coprinus comatus* suspension. Rats were sacrificed 24 hours after carbon tetrachloride administration [18]. Rats were narcotized with 25% solution of Urethane (Sigma Chemicals Co, St Louis, MO, USA), 4-5 mL/kg i.p. After loss of straightening reflex, animals were sacrificed by exsanguination. After killing, liver samples were taken for cross sections and homogenate preparation.

### 3.3. Cross sections

Tissue samples for cross sections were kept in 10% solution of formalin. The cross-sections were stained by a standard hematoxylin-eosin technique [19]. A Leica microscope combined with a Leica DC 100 photo camera was used for cross section analysis (magnification 400×). Liver homogenate was prepared from liver tissue (1 g) which was homogenized in a Potter homogenizer with TRIS-HCl sucrose in a ratio 1:3 at 4 ºC. The obtained homogenates were filtered and the biochemical parameters were determined.

### 3.4. Biochemical assays

Extent of lipid peroxidation, LPx, was determined after Buege and Aust [20], peroxidase (Px) activity was measured after Simon *et al.* [21] and the effect of catalase (CAT) after Beers and Sizer [22]. Glutathione peroxidase (GSH-Px) activity was evaluated as described in Chin *et al.* [23], xanthine oxidase (XOD) after Bergmayer [24], and reduced glutathione content (GSH) after Kapetanović and Mieyal [25]. The total protein content was determined after Gornall *et al.* [26].

### 3.5. Statistical analysis

Results of biochemical analyses are presented as the mean value±standard deviation (S.D.). The difference between control and test groups was analyzed using the Student t-test (significant difference at p ≤ 0.05 confidence level). 

## 4. Conclusions

According to the obtained results, it can be concluded that two screening doses of alloxan, which lead to diabetes development in rats, did not significantly influence the examined biochemical parameters of liver homogenate, nor the cytoarchitectonics of liver cross sections. Carbon tetrachloride acted as prooxidant and increased the intensity of lipid peroxidation in liver homogenate in a statistically significant manner and led to certain changes in liver cross sections as well. Seven day treatment with *Coprinus comatus* suspension had protective effects on liver homogenate biochemical parameters and liver tissue.
